# Cerebral Meningitis

**Published:** 1881-09

**Authors:** James Hutchinson

**Affiliations:** Maine


CHICAGO MEDICAL

JOURNAL & EXAMINER.

VOL. XLIII.—SEPTEMBER, 1881.—No. 3.

### Clinical Lectures.

ARTICLE I.

CEREBRAL MENINGITIS. A Clinical Lecture by JAMES HUTCHINSON, M.D., of Maine.

This woman is 31 years of age, and was admitted on the 4th of February, 1879. Her father died of consumption, and one of her sisters of paralysis, but the rest of her family history, as far as it can be obtained, is good. Her mother says that she has been subject to convulsions since she was three years old, and that she began to show evidence of scrofula when quite a young girl. She has had frequent abscesses situated upon her extremities and trunk, from each of which dead bone has been discharged. Her mother also states that when she was 14 years old, and was living on a plantation in Virginia, she was attacked by her master on one occasion when he returned home much intoxicated, and was almost killed. She succeeded in making her escape from him, when it was discovered that her right shoulder was dislocated and that there was a subluxation of the left knee joint. She was so badly beaten and bruised that she was obliged to keep to her bed for a year afterwards. Her knees, according to her mother's account, only began to swell after she was beaten. Since that time the convulsions, so-called, have been more frequent. Menstruation began at the age of 21, and has been very irregularly performed ever since. She has suffered much from dysmenorrhœa. The convulsions are much more frequent at the menstrual periods but are by no means confined to those times. In the winter of 1875–76, she had a succession of chills, followed by cough and expectoration, which was first tenacious and yellowish, but afterward muco-purulent and blood-stained. She had one or two slight hæmorrhages. She then suffered from headache, vertigo and faintness for about a week. Soon after this, while sitting at table, she fell to the floor unconscious, and remained so all day. She had no actual convulsion nor did she foam at the mouth. She was completely aphasic upon recovering consciousness. Her head was turned slightly to the left and rigidly held there. The left arm and leg were also rigid and powerless. The right hand was also slightly affected, and there was some rigidity of the right knee. There was also divergent strabismus of the right eye, with diplopia and flashes of light before the eyes. In one week the power of speech returned, and in the course of one month she had regained the power of motion in the right arm and leg and in the left arm. At this time the patient's bowels were constipated and there was retention of urine requiring catheterization. In the summer of 1876 she had anorexia, nausea and vomiting, and great pain in the abdomen which was very sensitive to pressure. She also had headache. About December, 1876, she had two copious hæmorrhages from the lungs. Soon afterward her articulation became imperfect, and in February, 1879, she had a violent convulsion which came on during sleep. On this occasion she foamed at the mouth and lacerated her tongue severely. She was unconscious for some hours, and upon recovering was again completely aphasic, and had paralysis of the muscles of deglutition, which lasted for one week and necessitated the use of nutritious enemata. There was no paralysis or rigidity of the muscles of the limbs or face, but her vision became much more indistinct than before. After this attack she was in bed about three months and during that time she was constipated and had occasional retention of urine. The pain in her abdomen also returned after this attack. In August, 1877, she was admitted to this hospital, suffering from abscess in the side. While in the hospital she had a convulsion in which she foamed at the mouth and bit her tongue. After this she had aphasia for a short time. Three months later she had a fourth convulsion, and has been confined to her bed almost ever since. The abdominal pain returned after each of her last convulsions, and upon admission is still present. She denies all venereal taint, though she has suffered from sore throat. She has had a vaginal discharge for a long time. Upon her second admission the patient is quite thin and her body and legs are covered with cicatrices, the seats of former abscesses, but upon the left leg are several shining superficial scars. There are also signs of old synovitis of the right knee, and the ligaments of the left knee are very lax, allowing great freedom of motion of the leg in every direction. The middle finger of the left hand has its distal articulation semi-dislocated. There is divergent strabismus of the right eye. There is some dnllness over the right chest posteriorly. The respiratory sounds are feeble and there is a trace of ægophony. The urine is acid; sp. gr., 1018; no albumen.

CLINICAL RECORD.

February 5. Ordered mist. gent. comp. f℥ss. t. d. Patient had an attack last night, which lasted but a short time, in which she became unconscious without any convulsion.

February 10. Ordered potass. iod. gr. x. t. d. and mist. gent. comp. dropped.

February 12. There is a dwindling of the right leg below the knee. The right calf measures ten and three-fourths inches, and the left calf twelve and a half inches.

February 13. Patient complains of pain upon micturition. Vaginal examination reveals a prolapse of the womb and considerable leucorrhœa. Ordered injection of alum (gr. xxx to Oj of water).

February 17. Ophthalmoscopic examination reveals atrophy of the discs, the result of former choking.

February 18. Considerable pain in abdomen.

February 19. Patient says that she passes blood by stool. Once or twice during the past two weeks she seemed to have lost the power of articulation for an hour or two at a time (she has never lost consciousness at these periods), and when she has regained speech has stated that she had severe pain over the region of the heart.

February 20. Patient is rapidly improving. The strabismus is much less marked.

February 21. Ordered ol. morrhuæ, f℥ss t. d. Iodide continued.

February 30. Convalescing rapidly.

---

POST-MORTEM EVIDENCES OF DISEASE IN CEREBRO-SPINAL MENINGITIS.—Rigor mortis marked. Posterior and lateral surfaces of body marked with livid spots of various sizes; also anterior surfaces of both arms and upper part of both forearms and knees exhibited little clusters of punctate spots, each of the size of the head of a small pin; some over the free border of the ribs on the right side. The peritoneum, and all the serous membranes exposed, presented a very deep, almost violet color. The pericardium contained a drachm of clear serum; membrane normal. The heart weighed three ounces and one-half; valves normal, right ventricle of auricle contained white clots; left ventricle contained fluid blood. The left pleural cavity contained some old adhesions at the base of the lung. The left lung weighed four and one-half ounces; hypostatic congestion of the lower lobe; crepitated throughout; diaphragmatic pleurisy. The right lung weighed five and one-half ounces, crepitated throughout. The liver weighed thirty ounces, was congested, and softer than normal. The right kidney weighed two ounces, the left two and one-half ounces, rather soft; cortex looked fatty, no irregularity in markings. The brain weighed two pounds and fourteen ounces; intensely congested, and in meshes of pia mater were collections of purulent material. The blood-vessels, after opening the dura mater of the cord, were found distended with blood, and in the meshes of the pia were collections of pus.—*N. Y. Med. Record.*

